# Retrospective analysis of outcomes following inferior vena cava (IVC) filter placement in a managed care population

**DOI:** 10.1007/s11239-017-1507-z

**Published:** 2017-05-26

**Authors:** Damian Everhart, Jamieson Vaccaro, Karen Worley, Teresa L. Rogstad, Mitchel Seleznick

**Affiliations:** 1 Centers for Medicare and Medicaid Services (CMS), Baltimore, MD USA; 2 Comprehensive Health Insights, Humana Inc., 515 West Market Street, Louisville, KY 40202 USA; 3 Humana Inc., 500 West Main Street, 14th Floor, Louisville, KY USA; 4 CarePlus Health Plans, Humana Inc., 11430 NW 20th Street, Suite 300, Miami, FL USA; 5 Centers for Medicare & Medicaid, c/o 1449 SW Ibis Street, Palm City, FL 34990 USA

**Keywords:** Surgery, Thromboembolism, Inferior vena cava filter, Utilization

## Abstract

The role of inferior vena cava filter (IVC) filters for prevention of pulmonary embolism (PE) is controversial. This study evaluated outcomes of IVC filter placement in a managed care population. This retrospective cohort study evaluated data for individuals with Humana healthcare coverage 2013–2014. The study population included 435 recipients of prophylactic IVC filters, 4376 recipients of therapeutic filters, and two control groups, each matched to filter recipients. Patients were followed for up to 2 years. Post-index anticoagulant use, mortality, filter removal, device-related complications, and all-cause utilization. Adjusted regression analyses showed a positive association between filter placement and anticoagulant use at 3 months: odds ratio (ORs) 3.403 (95% CI 1.912–6.059), prophylactic; OR, 1.356 (95% CI 1.164–1.58), therapeutic. Filters were removed in 15.67% of prophylactic and 5.69% of therapeutic filter cases. Complication rates were higher with prophylactic procedures than with therapeutic procedures and typically exceeded 2% in the prophylactic group. Each form of filter placement was associated with increases in all-cause hospitalization (regression coefficient 0.295 [95% CI 0.093–0.498], prophylactic; 0.673 [95% CI 0.547–0.798], therapeutic) and readmissions (OR 2.444 [95% CI 1.298–4.602], prophylactic; 2.074 [95% CI 1.644–2.616], therapeutic). IVC filter placement in this managed care population was associated with increased use of anticoagulants and greater healthcare utilization compared to controls, low rates of retrieval, and notable rates of device-related complications, with effects especially pronounced in assessments of prophylactic filters. These findings underscore the need for appropriate use of IVC filters.

**Electronic supplementary material:**

The online version of this article (doi:10.1007/s11239-017-1507-z) contains supplementary material, which is available to authorized users.

## Introduction

The annual U.S. incidence of venous thrombolytic embolism (VTE), including both deep vein thrombosis (DVT) and pulmonary embolism (PE), is 1 to 2 per 1000 persons, and approximately one-third will recur within 10 years [1]. IVC filters provide an alternative option for prevention of PE when anticoagulation therapy has failed or is contraindicated [2]. Indications for IVC filter are considered prophylactic in the absence of a history of VTE or therapeutic to prevent recurrence of VTE However, the ability of IVC filters to improve health outcomes remains uncertain. In the only two randomized controlled trials of IVC filters published to date, therapeutic filters were implanted as an add-on to anticoagulation [3–5]. Neither trial detected a statistically significant reduction in PE at 6 months. In the first trial, called the PREPIC trial, 8-year follow-up revealed a significant reduction in PE attributable to filter [4]. A recent systematic review identified 11 observational studies comparing IVC filter to no filter, with or without concomitant anticoagulation. Study results were mixed in terms of outcome measures and the direction of findings [6]. A more recent observational study (n = 688) evaluated IVC filter in patients with previous VTE and contraindication to anticoagulants and detected a reduction in PE-related death at 30 days [7].

The use of IVC filters varies widely by type of hospital, geographic location, and insurance status [8–10]. Utilization has grown dramatically following the introduction of retrievable filters in the early 2000s [2, 6, 11], particularly for prophylactic indications [11, 12]. IVC filter placement in the Medicare fee-for-service population increased 111.5% between 1999 and 2008 [13]. In addition, research has suggested that the placement of IVC filters often occurs in patients who do not have the indications recommended by published guidelines at the time of filter placement [14–16].

Of potential concern is the low rate of filter removal despite the growing use of retrievable filters. According to one estimate, 50% of all newly placed filters in 2010 were retrievable and the authors projected an increase to 75% by 2012 [11]. However, Medicare fee-for-service data reflect overall removal rates of an estimated 1–5% in 2008 [13]. A 2011 systematic review reported an average retrieval rate of 34% across 37 studies of retrievable filters [2], and more recent institutional studies of retrievable filters have reported removal rates of 9–63%, often with the conclusion that removal rates were lower than they should be [16–20]. In one study 25% of 978 patients with IVC filters were discharged on anticoagulants, suggesting transient contraindications, but filters were removed in only 8.5% of patients [16].

These trends raise safety concerns because of the risk of device-related complications and DVT attributable to the inserted filter [11, 21]. In the PREPIC trial the incidence of recurrent symptomatic DVT at 8 years was approximately 50% greater in the IVC filter group [4]. In 2010 the FDA urged physicians to consider retrieval as soon as protection from PE is no longer needed because of the 921 device-related adverse events reported since 2005 and findings from an FDA literature view [22].

The objective of this study was to assess the safety of IVC filter placement by comparing outcomes between patients who underwent IVC filter placement for either prophylactic or therapeutic purposes and potential candidates for IVC filter placement who did not undergo the procedure.

## Methods

This two-part retrospective cohort study with matched control groups was based on claims data. The study sample included individuals with a commercial or Medicare Advantage Prescription Drug (MAPD) plan with Humana, a health and wellness company that insured more than 3.83 million individuals under commercial or MAPD plans in 2014 [23]. See the Appendices II.A–II.H (Online Resources) for all diagnosis and procedure codes used to identify participants and define variables. The study received Institutional Review Board approval from Schulman IRB, with a waiver of Health Insurance Portability and Accountability Act (HIP AA) authorization.

### Participants

General patient inclusion criteria included participation in a commercial plan or MAPD plan with prescription and medical coverage during the interval January 1, 2013 to December 31, 2014 (identification period), lack of VTE due to sepsis or pregnancy during the identification period, and age 22–89 years. Two IVC filter groups were selected based on Current Procedural Terminology (CPT)-4 code 37,191: (1) a Prophylactic IVC Filter Group without a PE or DVT diagnosis and (2) a Therapeutic IVC Filter Group with a diagnosis of PE or DVT during the identification period and prior to filter placement. The presence of a PE or DVT diagnosis was determined by International Classification of Disease (ICD)-9 codes (Appendix II-A). Individuals who met inclusion criteria for both IVC filter groups were excluded so that the two groups were mutually exclusive. The initial occurrence of a paid claim for filter placement was considered the index date for the two filter groups.

Possible controls for the Prophylactic IVC Filter Group were individuals who met general inclusion criteria, had no PE or DVT diagnosis, and underwent surgery without filter placement. They were matched 1:1 to cases according to type of surgery (same CPT-4 code ± 30 days of the case patient’s surgery), age, and gender, with the surgery date serving as the index date. Possible controls for the Therapeutic IVC Filter Group were individuals who met general inclusion criteria and had any claim with a diagnosis code for PE or DVT during the identification period but no IVC filter placement. They were matched 1:1 according to date of PE/DVT diagnosis (diagnosis ± 30 days of the case patient’s filter placement), age, and gender, with the filter placement date of the matched filter recipient serving as the index date.

### Outcome measures

Anticoagulant use during the post-index period was evaluated under the assumption that if patients were expected to benefit from anticoagulants, they may not have been appropriate candidates for IVC filter placement; and was measured as a continuous variable (total number of anticoagulant prescription fills, normalized to a 30-day supply) assessed at last follow-up and as a categorical variable for any use at 3, 6, 12, and 24 months. See Appendix II-G for a list of the anticoagulants considered. Mortality was assessed for the interval between index date and the time of last follow-up. Mortality information was derived from the Social Security Administration death files and available only for MAPD participants (96% of the combined study samples, with relatively even distribution between filter and control groups; see Table 1). Last follow-up, ≤2.5 years, occurred at disenrollment, death, or study end (June 30, 2015).

Vascular device-related complications at any time during follow-up were measured categorically according to prespecified ICD-9-CM codes (see Appendix II-H). These codes were chosen based on reports in the literature of filter migration and fracture [12, 17, 24]. IVC filter removal was measured categorically according to a paid claim for CPT-4 code 37193.

Post-index all-cause healthcare utilization was assessed as a proxy for general morbidity. Hospitalizations, readmissions, emergency department (ED) visits, and physician office visits were measured as categorical (0 or ≥1) and as continuous variables (number of encounters). Measurement was limited to 6 months since all-cause utilization would be increasingly less likely to be attributable to filter placement over time.

### Covariates and other measures of interest

Baseline demographic variables included age, gender, geographic region, race/ethnicity, and low income subsidy (LIS) status. Geographic residence was categorized according to U.S. Census Bureau regions (Northeast, Midwest, South, and West). Race/ethnicity information was available only for MAPD participants and was obtained from linked socio-demographic data and categorized as follows: White, African-American/Black, Hispanic, or Other.

Prior or concurrent comorbidities of interest were identified by the presence ICD-9-CM codes during the 6-month pre-index period (see Appendix II-A). The RxRisk-V comorbidity score, which is based on pharmacy claims [25–27], provided a composite measure of clinical risk. Baseline bleeding risk was measured by a modified HAS-BLED score, a validated tool for calculating 1-year major bleeding risk [28]. Previous healthcare utilization was identified for the 6-month pre-index period as a proxy measure for baseline morbidity.

### Statistical analysis

All analyses were conducted using SAS Enterprise Guide 5.1. The a priori alpha level for all inferential analyses was set at 0.05. Differences in baseline characteristics for unmatched variables and unadjusted comparisons of outcomes were tested by McNemar tests for categorical variables and paired* t* tests (normal distribution) or Wilcoxon signed rank tests (nonnormal distribution) for continuous variables.

Anticoagulant use was compared between filter and control groups. It was treated as a categorical variable in unadjusted analysis. Adjusted analysis was performed with a generalized linear [regression] model (GLM) (continuous variable) accounting for matched pairs and with a conditional logistic regression (categorical variable) model. In addition to filter placement, model covariates included race/ethnicity (white as reference); RxRsk-V score; number of pre-index physician office visits, inpatient admissions, and emergency department (ED) visits; geographic region (non-South as reference); HAS-BLED Risk Score; 11 predefined clinical events or chronic disease diagnoses; and pre-index anticoagulation use (continuous variable). The comorbid conditions were acute myocardial infarction, cardiomyopathy, cancer, coronary artery disease, fracture of the lower extremity, ischemic stroke, head injury, transient ischemic attack, heart failure, hypertension, and peripheral artery disease (see Appendices II-B through II-E).

Mortality was compared between filter groups and control groups in unadjusted analysis and through a Cox Proportional Hazards model, using Sandwich Variance Estimation to account for matched pairs. Model covariates were the same as those included in the models for anticoagulant use, with the exception of pre-index anticoagulant use. Filter removal was compared between prophylactic and therapeutic filter groups in unadjusted analysis and through a Cox Proportional Hazards model, with the intention to use the same covariates as those included in the mortality model. Complications were not compared statistically because of low frequencies.

Only individuals with continuous enrollment in the 6-month *post*-index period were included in the assessment of healthcare utilization. Comparisons were made between filter and control groups in an unadjusted analysis and with use of GLM (physician office visits, inpatient admissions, ED visits) accounting for matched pairs and conditional logistic regression (readmission) models. Model covariates were the same as those used in the mortality models.

## Results

**Fig. 1:**
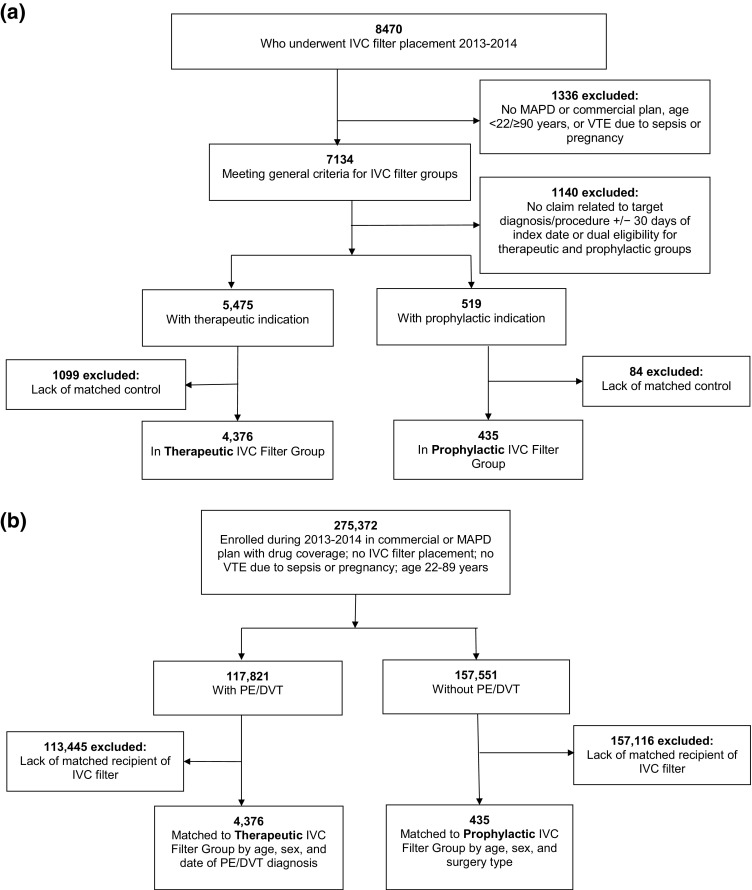
**a** Participant flow diagram, IVC filter groups. **b** Participant flow diagram, control groups

As shown in Fig. 1a, b, application of inclusion and exclusion criteria and matching resulted in 435 patients each in the Prophylactic IVC Filter Group and the Prophylactic Control Group, and 4376 patients each in the Therapeutic IVC Filter Group and the Therapeutic Control Group. Of the 7134 individuals in the two IVC filter groups, 6510 (91.3%) were in a MAPD plan and 624 (8.7%) were in a commercial plan. Other baseline characteristics of the final study samples are presented in Table 1. Mean age and sex distribution were very similar between each set of filter and control groups. Filter groups had significantly greater comorbidity according to individual diagnoses, the pharmacy-based RxRisk-V comorbidity score, bleeding risk (HAS-BLED score), and pre-index healthcare utilization. Mean follow-up in days for all individuals in each group was 200.9 (therapeutic filter), 271.2 (control, therapeutic), 257.2 (prophylactic filter), and 275.5 (control, prophylactic).

**Table 1:** Baseline characteristics

Variable	Prophylactic		Therapeutic	
	IVC filter (n = 435)	Control (n = 435)	IVC filter (n = 4376)	Control (n = 4376)
Age in years, mean (SD)	69.46 (10.26)	69.46 (10.26)	72.86 (9.28)	72.86 (9.28)
Male Sex, n (%)^a^	203 (46.67%)	203 (46.67%)	2154 (49.22%)	2154 (49.22%)
Race/ethnicity, n (%)^b^				
Black	51 (12.69%)	34 (8.81%)	688 (16.80%)	565 (13.73%)
Hispanic	4 (1.00%)	4 (1.04%)	61 (1.49%)	61 (1.48%)
White	337 (83.83%)	335 (86.79%)	3,273 (79.91%)	(3,412) 82.92%
Other	10 (2.49%)	13 (3.37%)	74 (1.8%)	(77) 1.87%
Geographic region, n (%)				
Northeast	9 (2.07%)	7 (1.61%)	88 (2.01%)	75 (1.71%)
Midwest	108 (24.83%)	104 (23.91%)	1,061 (24.26%)	1,056 (24.15%)
South	290 (66.67%)	283 (65.06%)	2,859 (65.36%)	2,836 (64.84%)
West	28 (6.44%)	41 (9.43%)	368 (8.41%)	407 (9.30%)
Low Income Subsidy, n (%)	8 (1.84%)	5 (1.15%)	106 (2.42%)	41 (0.94%)
Plan population				
MAPD	403 (92.64%)	386 (88.74%)*	4,097 (93.62%)	4,117 (94.08%)
Commercial	32 (7.36%)	49 (11.26%)	279 (6.38%)	259 (5.92%)
Comorbidities				
Acute myocardial infarction	25 (5.75%)	5/31%	481 (11.03%)	143 (3.48%)^‡^
Cardiomyopathy	21 (4.83%)	4.395	410 (9.40%)	267 (6.51%)^‡^
Cancer	125 (23.74%)	21.48%*	1780 (40.81%)	974 (23.73%)^‡^
Coronary artery disease	159 (36.55%)	32.56%	1649 (37.80%)	1,231 (30.00%)^‡^
Fracture of lower extremity	46 (10.57%)	4.85%*	222 (5.09%)	66 (1.61%)^‡^
Ischemic stroke	28 (6.44%)	1.85%^†^	393 (9.01%)	149 (3.63%)^‡^
Head injury	12 (2.76%)	0.23%*	39 (0.89%)	4 (0.10%)^‡^
Transient Ischemic attack	25 (5.75%)	2.31%*	275 (6.30%)	138 (3.36%)^‡^
Heart failure	111 (25.52%)	13.39%^‡^	1,430 (32.78%)	805 (19.62%)^‡^
Hypertension	372 (85.52%)	75.06%^‡^	3,709 (85.03%)	2,996 (73.00%)^‡^
Peripheral artery disease	113 (25.98%)	19.40%*	1,129 (25.88%)	805 (19.62%)^‡^
HAS-BLED risk score n (%)				
Low risk (0–1)	54 (12.41%)	113 (26.10%)^‡^ (global P)	339 (7.76%)	1,140 (27.08%)^‡^ (global P)
Intermediate risk (2)	73 (16.78%)	90 (20.79%)	587 (13.44%)	1,051 (24.97%)
High risk (≥3)	308 (70.805)	230 (53.12%)	3,441 (78.80%)	2,018 (47.94%)
Rx-Risk-V Score, mean (SD)	7 (3)	6 (3)^‡^	7 (3)	6 (3)^‡^
Pre-Index^c^ Utilization, mean number of encounters (SD)				
Physician office visits	26 (22)	18 (15)^‡^	21 (21)	15 (16)^‡^
Hospital admissions	2 (2)	1 (1)^‡^	2 (2)	11^‡^
Emergency department visits	2 (2)	1 (1)^‡^	2 (2)	1 (2)^‡^

*MAPD* medicare advantage prescription drug

*P < 0.05; ^†^P < 0.01; ^‡^P < 0.001; filter versus control or global P, filter versus control across strata

^a^The denominator varies slightly across cells within and between groups because of small differences in missing data, primarily due to lack of socioeconomic data for individuals in commercial plans

^b^Race/ethnicity information was available only for individuals under Medicare Advantage Prescription Drug plans; thus, individuals with commercial plans were not included in these counts

^c^The pre-index period was 12 months

Adjusted analysis found that prophylactic filter placement was associated with an increase in the number of 30-day anticoagulant fills at last follow-up. Logistic regression likewise demonstrated that the odds of anticoagulation use increased with prophylactic filter placement by approximately threefold, whether assessed at 3, 6, 12, or 24 months post-index. Positive (though smaller) associations between therapeutic filter placement and subsequent anticoagulant were also observed. See Table 2.

**Table 2:** Post-index anticoagulant use

Measure interval	Prophylactic			Therapeutic		
	IVC filter (n = 435)	Control (n = 435)	P value	IVC filter (n = 4376)	Control (n = 4376)	P value
Mean(SD) normalized number of 30-day anticoagulant fills						
At last follow-up	4(6)	1(2)	<0.001	4(6)	4(6)	0.45
Additional adjusted normalized 30-day anticoagulant refills (95% CI) attributable to IVC filter^a^						
At last follow-up	1.58 (1.114–2.046)		<0.001	0.3903 (0.2827–0.4979)		<0.001
Number of patients (% of original sample), with anticoagulant use^b,c^						
3 months	209 (48.05%)	91 (20.92%)	<0.001	1836 (41.96%)	1905 (43.53%)	0.14
6 months	223 (51.26%)	99 (22.76%)	<0.001	1960 (44.79%)	2047 (46.78%)	0.06
12 months	236 (54.25%)	108 (24.83%)	<0.001	2015 (46.05%)	2110 (48.22%)	0.04
24 months	239 (54.94%)	114 (26.21%)	<0.001	2041 (46.64%)	2145 (49.02%)	0.03
Adjusted relative likelihood of anticoagulant sse, OR (95% CI)^c,d^						
3 months	3.403 (1.912–6.059)		<0.001	1.356 (1.164–1.58)		<0.001
6 months	3.771 (1.903–7.474)		<0.001	1.316 (1.127–1.536)		<0.001
12 months	3.753 (2.012–7.002)		<0.001	1.274 (1.091–1.487)		0.0022
24 months	3.15 (1.727–5.744)		<0.001	1.257 (1.076–1.467)		0.0038

*IVC* inferior vena cava

^a^Numerical results represent the coefficient for IVC filter in a generalized linear [regression] model (GLM). In addition to filter placement, model covariates included race/ethnicity (white as reference value); RxRsk-V score; number of pre-index physician office visits, inpatient admissions, and emergency department visits; geographic region (non-South as reference value); HAS-BLED Risk Score; 11 predefined clinical events or chronic disease diagnoses; and pre-index anticoagulation use

^b^Because of attrition due to death and disenrollment, the number of individuals with available data diminished as the measurement interval increased. Thus, the percentages presented here are smaller than they would be if calculated with the actual number of remaining patients for each time interval and most likely reflect an underestimate of the increasing use of anticoagulants

^c^Intervals were defined as 0–91 days (3 months), 0–181 days (6 months), 0–365 days (12 months), and 0–730 days (24 months)

^d^Logistic regression (binary variable), using the same covariates as those included in the GLM model

Mortality was 22.76 versus 11.03% (P < 0.001), filter versus control group, in the prophylactic population. After adjustment for covariates and varying lengths of follow-up, the difference was no longer statistically significant. Unadjusted rates were 44% versus 18% (P < 0.0001) in the therapeutic population, and adjusted analysis resulted in a statistically significant association favoring controls. See Table 3 for the HR values for all covariates.

**Table 3:** Mortality, inferior vena cava filter versus control

Variable	Prophylactic^a^	Therapeutic^a^
	Hazard ratio (95% CI)	Hazard ratio (95% CI)
Index group (reference = control group)	1.359 (0.861–2.145)	1.893 (1.7–2.107)^‡^
Race/ethnicity (reference = White)	0.886 (0.477–1.646)	0.918 (0.821–1.025)
RxRisk-V score	0.993 (0.913–1.08)	1.004 (0.988–1.022)
Pre-index utilization		
Physician office visits	0.995 (0.986–1.003)	1.005 (1.003–1.007)^‡^
Hospital admissions	1.009 (0.901–1.13)	1.059 (1.031–1.087)^‡^
Emergency department visits	1.164 (1.071–1.266)^‡^	1.033 (1.009–1.057)^†^
Geographic region (reference = south)	0.96 (0.627–1.469)	1.083 (0.984–1.192)
HAS-BLED risk score	1.237 (1.015–1.507)*	1.173 (1.28–1.22)^‡^
Comorbidities of interest		
Acute myocardial infarction	1.315 (0.673–2.57)	1.073 (0.921–1.249)
Cardiomyopathy	0.799 (0.347–1.84)	1.189 (1.02–1.386)*
Cancer	2.100 (1.384–3.186)^‡^	2.229 (2.032–2.445)^‡^
Coronary artery disease	0.907 (0.558–1.472)	0.983 (0.88–1.087)
Fracture of the lower extremity	1.05 (0.568–1.941)	1.258 (0.991–1.598)
Ischemic stroke	2.007 (0.939–4.289)	1.114 (0.937–1.325)
Head injury	7.115 (1.57–32.238)*	0.816 (0.4–1.665)
Transient ischemic attack	0.99 (0.479–2.044)	0.99 (0.824–1.189)
Heart failure	1.654 (1–2.735)	1.43 (1.288–1.587)^‡^
Hypertension	0.893 (0.454–1.755)	0.744 (0.645–0.857)^‡^
Peripheral artery disease	1.196 (0.774–1.85)	1.045 (0.942–1.158)

*P < 0.05; ^†^P < 0.01; ^‡^P < 0.001

^a^Mortality information was available only for individuals under Medicare Advantage Prescription Drug plans (6510 individuals across the two filter groups)

Filters were removed in 68 (15.67%) of the prophylactic filter recipients and in 249 (5.69%) of the therapeutic filter recipients. Head injury could not be included as a model covariate because of missing data. After adjustment for the other confounders and for varying follow-up time, filter removal was approximately half as likely following therapeutic filter placement compared with prophylactic filter placement. See Table 4. Table 5 shows complication rates of <1–5%.

**Table 4:** Filter removal, therapeutic versus prophylactic inferior vena cava filter placement

Variable	Hazard ratio (95% CI)	P value
Index group (reference = prophylactic)	0.479 (0.35–0.656)	<0.001
Race/ethnicity (reference = white)^a^	1.558 (1.174–2.067)	0.002
RxRisk-V score	0.98 (0.931 − 0.131)	0.44
Pre-Index utilization		
Physician office visits	1.006 (0.999–1.013)	0.09
Hospital admissions	0.871 (0.763–0.995)	0.04
Emergency department visits	0.818 (0.731–0.916)	<0.001
Geographic region (reference = south)	2.389 (1.844–3.096)	<0.001
HAS-BLED risk score	0.823 (0.737–0.919)	<0.001
Comorbidities of interest		
Acute myocardial infarction	1.073 (0.626–1.839)	0.80
Cardiomyopathy	1.566 (0.892–2.75)	0.12
Cancer	0.814 (0.607–1.092)	0.17
Coronary artery disease	0.949 (0.696–1.293)	0.74
Fracture of the lower extremity	0.866 (0.467–1.606)	0.65
Ischemic Stroke	0.862 (0.429–1.735)	0.68
Transient ischemic attack	1.052 (0.528–2.098)	0.88
Heart failure	0.598 (0.401–0.89)	0.01
Hypertension	1.169 (0.814–1.681)	0.40
Peripheral artery disease	0.682 (0.472–0.985)	0.04

^a^Race/ethnicity information was available only for individuals under Medicare Advantage Prescription Drug plans (91.3% of overall study group)

**Table 5:** Device-related complications

	Prophylactic (n = 435)	Therapeuctic (n = 4376)
Vascular complication, n (%)		
Mechanical complication (ICD-9-CM^1^ 996.1)	9 (2.07%)	89 (2.03%)
Infection or inflammatory reaction (ICD-9-CM 996.62)	10 (2.30%)	35 (0.80%)
Other complications due to a vascular device (ICD-9-CM 996.74)	22 (5.07%)	82 (1.87%)
Other vascular complications of medical care, not elsewhere classified (ICD-9-CM 999.2)	4 (0.92%)	9 (0.21%)
Days to first complication, mean(SD) and median[IQR]	62 (76) 33 [9–83]	96 (136) 37 [7–112]

*ICD* international classification of disease

Prophylactic and therapeutic IVC filter placement were both associated with increases in healthcare utilization. Adjusted analyses showed a significant increase in the number of per-patient post-index hospitalizations and ED visits associated with prophylactic filter placement, as well as a significant increase in number of per-person hospitalizations associated with therapeutic filter placement. Compared with control groups, both prophylactic and therapeutic groups were associated with more than twice the odds of readmission. No effect on physician office visits was observed. See Table 6.

**Table 6:** All-cause 6-month healthcare utilization

Variable	Prophylactic (n = 713)^a^		Therapeutic (n = 6799)^a^	
	Comparison with control	P value	Comparison with control	P value
Impact on number of encounters, GLM model parameter^b^				
Physician office visits	0.0392 (−0.1313–0.2097)	0.65	−0.0557 (−0.144–0.0327)	0.22
Inpatient admissions	0.295 (0.093–0.498)	0.0042	0.673 (0.5473–0.7976)	<0.001
Emergency department visits	0.4214 (0.0729–0.7699)	0.02	−0.0846 (−0.2202–0.051)	0.22
Relative likelihood, OR^b^				
Readmission	2.444 (1.298–4.602)	0.0056	2.074 (1.644–2.616)	<0.001

^a^Number of participants with 6 months of continuous post-index enrollment

^b^In addition to filter placement, model covariates included race/ethnicity (white as reference value); RxRsk-V score; number of pre-index physician office visits, inpatient admissions, and emergency department (ED) visits; geographic region (non-South as reference value); HAS-BLED Risk Score; and 11 predefined clinical events or chronic disease diagnoses

## Discussion

This study of a managed care population adds to the small body of studies reporting real-world outcomes for IVC filter placement. Results for post-index anticoagulant use were unanticipated. Although contraindication to, or failure of, anticoagulation therapy is the chief indication for IVC filter, 48% of recipients of a prophylactic filter and 42% of recipients of therapeutic filters received anticoagulants in the 3-month period post-index. These findings are even more surprising than those reported by Sarosiek et al. [16], who found that 25% of IVC filter recipients at a large academic hospital were discharged on some form of anticoagulant therapy. Furthermore, anticoagulation use in the present study was greater in the filter groups than in the control groups after adjusting for confounders, including prior use of anticoagulants, with the difference persisting up to 2 years. The adjusted association was especially strong in the prophylactic subpopulation even though it is likely that most of the individuals in the prophylactic filter group had only a transient contraindication to anticoagulants. Studies have documented the increased risk of DVT associated with implanted IVC filters [11, 21]. Thus, the greater use of anticoagulants in both filter groups compared with control groups may in many cases reflect concern over the risk posed by the filters themselves. It is also possible that in some cases IVC filters were being used to augment anticoagulant therapy. Practice guidelines do recommend resumption of anticoagulants in patients with filters after resolution of contraindications to anticoagulants or bleeding complications [29], but as noted in the introduction to this article, evidence is sparse with respect to the ability of filters, as add-on therapy, to reduce the incidence of PE.

Also noteworthy were this study’s findings that only a small percentage of filters were removed, 15.67% in the Prophylactic IVC Filter Group and 5.69% in the Therapeutic IVC Filter Group. These rates are better than the overall estimates of 1.2–5.1% reported by Duszak et al. [13] for Medicare claims in 2008. It was not possible to determine how many of the filters were designed to be removed, but published estimates suggest that most filters implanted in recent years would be retrievable [11]. Although considerably higher rates of removal of retrievable filters have been reported by some academic centers [18, 19, 30], other academic centers have reported rates around 9–14% [16, 17, 20]. One institution reported an improvement in retrieval rate from 63 to 100% with the implementation of a clinical pathway [18], suggesting that failure to retrieve at that institution had been due primarily to noncompliance with best practices. Similarly, evaluation of an educational campaign conducted across hospitals in a single region found that retrieval attempts increased from 38.9 to 54.0% (P = 0.0006) [30]. Given the reported success of IVC filter clinics [31, 32], routine post-implantation monitoring of patients may be required to assure that filters are removed as soon as is feasible.

Filter removal in the present study was considerably more likely in regions other than the South, which may reflect geographic practice variations. The greater concentration of the study population in the South compared with other regions thus contributed to the low rate of retrieval in the present study. The lower likelihood of filter removal following therapeutic versus prophylactic filter placement (adjusted HR 0.479; 95% CI 0.350–0.656) may reflect greater concern regarding ongoing risk of VTE in patients who have received filters for therapeutic reasons. The impact of IVC filter placement on mortality remains unknown. This analysis showed no improvement in mortality outcomes, and in contrast suggested that mortality increased with therapeutic filter placement. However, results must be interpreted with caution since the filter groups, compared with control groups, had greater baseline morbidity. Other observational research has reported a nonsignificant *reduction* in all-cause mortality and a significant reduction in PE-related death at 30 days attributable to filter placement in patients known to have an absolute or relative contraindication to anticoagulants [7].

Mechanical, device-related complication rates (approximately 2%) in the present study population were consistent with those reported in prior studies. A retrospective chart review revealed 10 instances of filter migration in a series of 952 patients undergoing therapeutic filter placement at a trauma center (1% incidence). The authors noted that the lack of standardized follow-up imaging may have obscured some cases of filter migration [16]. Data from a chart review at another institution yielded a 1.5% incidence of filter migration or tilt [24]. The 2% mechanical complication rate reported in the present study may have included other specific complications in addition to migration or tilt, given the use of ICD-9-CM codes for complications, which are more comprehensive than specific information often recorded in medical charts. Low retrieval rates may contribute to complication rates, as noted by Sariosek et al. [16], who observed a retrieval rate very similar to the rate reported for the present study. Since complications can occur during filter removal, further research is needed to assess the relative harms and benefits of filter removal. All-cause hospital- and ED-related measures of utilization in the 6-month post-index period were generally greater in IVC filter groups compared to control groups, which may reflect differences in baseline morbidity and/or adverse events related to filter placement.

Clinical guidelines published by the American College of Chest Physicians (ACCP), the Society of Interventional Radiology (SIR), and the American Heart Association (AHA) generally support a consideration of IVC filters in patients with documented DVT or PE and contraindications to anticoagulation therapy, failure of anticoagulation therapy, or poor cardiopulmonary reserve [21, 29, 33]. The ACCP and SIR advise considering prophylactic use of IVC filters in limited situations [33–35]. The American College of Physicians (ACP) and the American Association of Orthopaedic Surgeons contend that no evidence-based recommendations regarding IVC filter placement are possible, and the American Society of Hematology recommends against routine use of IVC filters in its Choosing Wisely^®^ list [36, 37]. See Appendix I (Online Resources) for more detail.

Certain limitations should be considered when interpreting the results of this study. Results may reflect a bias against the treatment groups since baseline morbidity and bleeding risk were substantially greater in IVC filter treatment groups compared with control groups. Although confounding variables were included in regression models, residual confounding is possible, given the magnitude of known baseline differences. The comparability of filter and control groups is also somewhat uncertain since it was not possible to ascertain from claims data whether individuals met guidelines-supported criteria for filter placement. The assessment of anticoagulant use following prophylactic filter placement may be biased because of less follow-up data for the filter group (mean 201, median 129 days) than for the control group (mean 271, median 231), but the direction of bias cannot be known. Although the codes used to identify device-related complications are not specific to IVC filters, it seems unlikely that a substantial number of study participants would have received vascular implantations in addition to IVC filter during the study period. Limitations common to errors in claims coding may have affected outcome measurement and the accuracy of the regression models. Results may not be generalizable to other managed care populations or to a general U.S. population. Furthermore, the West and Northeast regions of the United States were underrepresented due to Humana’s relatively small number of patients in those regions.

In summary, IVC filter placement in this managed care population was associated with increased use of anticoagulants, particularly in patients with prophylactic filters, with low rates of retrieval, and with increased hospital- and ED-related utilization. Notable rates of device-related complications were recorded. The ability of IVC filter placement to reduce all-cause mortality was not supported by study results. These findings underscore the need for appropriate use of IVC filters and can be used to guide physician training and reinforce compliance with clinical guidelines. Additional studies are needed to resolve inconsistencies in findings, better define subpopulations likely to benefit from filter placement, identify the relative safety of different devices, elucidate the reasons for anticoagulation use following filter placement and for low filter retrieval, and measure the risks associated with failure to retrieve filters.

## Electronic supplementary material

Below is the link to the electronic supplementary material.

Supplementary material 1 (DOCX 38 KB)
