# Supplementary material for: Retrospective analysis of outcomes following inferior vena cava (IVC) filter placement in a managed care population
Source: J Thromb Thrombolysis. 2017 May 26;44(2):179–89. doi: 10.1007/s11239-017-1507-z (PMC5522518; doi:10.1007/s11239-017-1507-z)
Supplement: Supplementary file 1 — Supplementary material 1 (DOCX 38 KB) [file 11239_2017_1507_MOESM1_ESM.docx]

**Title:** Retrospective Analysis of Outcomes Following Inferior Vena Cava (IVC) Filter Placement in a Managed Care Population

**Journal:** Journal of Thrombosis and Thrombolysis

**Authors:** Damian Everhart, PhD; Jamieson Vaccaro, MA; Karen Worley, PhD; Teresa L. Rogstad, MPH; Mitchel Seleznick, MD

**Corresponding Author:** Damian Everhart, RN, PHD; current affiliation, Centers for Medicare & Medicaid; damianeverhart@gmail.com

**Appendices**

**APPENDIX I. Recommendations from Major Practice Guidelines Regarding IVC Filter Placement**

| **Organization** | **Prophylactic Indications** | | **Therapeutic Indications** | | |
| --- | --- | --- | --- | --- | --- |
|  | **In Favor** | **Against** | **In Favor** | | **Against** |
| **American College of Chest Physicians**^1-3^ | Suggested in orthopedic surgery over no thromboprophylaxis in patients with increased bleeding risk or with contraindications to both pharmacologic and mechanical thromboprophylaxis. | General and abdominal-pelvic surgery  Trauma surgery | In patients with acute proximal DVT of the leg and contraindication to anticoagulation.  In patients with acute PE and contraindications to anticoagulation | | In addition to anticoagulants in patients with acute DVT of the leg  In patients with acute PE being treated with anticoagulants |
| **Society of Interventional Radiology (with review by the American College of Radiology**^4^ | Severe trauma, closed head injury, spinal cord injury, multiple long-bone or pelvic fractures, high risk (e.g., immobilized or in an intensive care unit) |  | PE or DVT and one of the following: contraindication to/complication of/ failure of anticoagulation; recurrent PE or propagation/progression of DVT during therapy; inability to achieve/maintain adequate anticoagulation; massive PE with residual DVT and risk for further PE, free-floating iliofemoral or IVC thrombus; severe cardiopulmonary disease and DVT | |  |
| **American Heart Association^5^** |  | | In adult patients with confirmed acute PE or proximal DVT with contraindications to anticoagulation or with active bleeding  In patients with recurrent DVT or acute PE despite therapeutic anticoagulation  To be considered in patients with acute PE and very poor cardiopulmonary reserve | | Routine use in treatment of ilio-femoral DVT |
| **American Association of Orthopaedic Surgeons**^6^ | Cannot recommend for or against in patients undergoing elective hip and knee arthroplasty who have contraindication to anticoagulation and/or known VTE. | | | | |
| **Society of Vascular Surgeons**^7^ |  | | Consider balance of harms and benefits in patients undergoing pharmacomechanical thrombolysis and in those with thrombus extending into IVC or who have markedly limited cardiopulmonary reserve | Routine use in conjunction with catheter-directed pharmacologic thrombolysis of iliofemoral venous segments | |
| **American College of Physicians**^8^ | There is insufficient evidence to make a recommendation. | | | | |
| **American Society of Hematology^9^** |  | |  | Do not use inferior vena cava filters routinely in patients with acute venous thromboembolism. | |

**Key:** DVT, deep vein thrombosis; IVC, inferior vena cava; PE, pulmonary embolism; VTE, venous thromboembolism

1. Falck-Ytter Y, Francis CW, Johanson NA, et al. Prevention of VTE in orthopedic surgery patients: Antithrombotic Therapy and Prevention of Thrombosis, 9th ed: American College of Chest Physicians Evidence-Based Clinical Practice Guidelines. Chest. 2012;141(2 Suppl):e278S-325S.
2. Gould MK, Garcia DA, Wren SM, et al. Prevention of VTE in nonorthopedic surgical patients: Antithrombotic Therapy and Prevention of Thrombosis, 9th ed: American College of Chest Physicians Evidence-Based Clinical Practice Guidelines. Chest. 2012;141(2 Suppl):e227S-277S.
3. Kearon C, Akl EA, Comerota AJ, et al. Antithrombotic therapy for VTE disease: Antithrombotic Therapy and Prevention of Thrombosis, 9th ed: American College of Chest Physicians Evidence-Based Clinical Practice Guidelines. Chest. 2012;141(2 Suppl):e419S-494S.
4. Vedantham S, Sista AK, Klein SJ, et al. Quality improvement guidelines for the treatment of lower-extremity deep vein thrombosis with use of endovascular thrombus removal. Journal of vascular and interventional radiology : JVIR. 2014;25(9):1317-1325.
5. Jaff MR, McMurtry MS, Archer SL, et al. Management of massive and submassive pulmonary embolism, iliofemoral deep vein thrombosis, and chronic thromboembolic pulmonary hypertension: a scientific statement from the American Heart Association. Circulation. 2011;123(16):1788-1830.
6. Jacobs JJ, Mont MA, Bozic KJ, et al. American Academy of Orthopaedic Surgeons clinical practice guideline on: preventing venous thromboembolic disease in patients undergoing elective hip and knee arthroplasty. J Bone Joint Surg Am. 2012;94(8):746-747.
7. Meissner MH, Gloviczki P, Comerota AJ, et al. Early thrombus removal strategies for acute deep venous thrombosis: clinical practice guidelines of the Society for Vascular Surgery and the American Venous Forum. J Vasc Surg. 2012;55(5):1449-1462.
8. Snow V, Qaseem A, Barry P, et al. Management of venous thromboembolism: a clinical practice guideline from the American College of Physicians and the American Academy of Family Physicians. Ann Fam Med. 2007;5(1):74-80.
9. Hicks LK, Bering H, Carson KR, et al. The ASH Choosing Wisely(R) campaign: five hematologic tests and treatments to question. Blood. 2013;122(24):3879-3883

**APPENDIX II. Diagnostic and Procedure Codes Used for Selecting Patients and Measuring Outcomes**

The diagnosis codes listed in the following tables were taken from the International classification of Diseases, Ninth Revision, Clinical Modification (ICD-9-CM).

**Appendix II-A. Included and Excluded ICD Codes for Identifying Patients with a History of Deep Vein Thrombosis (DVT) or Pulmonary Edema (PE)**

| **Included^1^** | **Excluded** |
| --- | --- |
| 451 Phlebitis and thrombophlebitis  451.1x Deep veins of lower extremities  451.2 Lower extremities, unspecified  451.81 Iliac vein  451.83 Deep veins, upper extremity  451.84 Unspecified veins, upper extremity  451.9 Unspecific site/not otherwise specified  453 Other venous embolism and thrombosis  453.2 Inferior vena cava  453.40 Lower extremities, not otherwise specified  453.41 Lower extremities, proximal  453.42 Lower extremities, distal  453.8x Other specified veins  453.9 Other unspecified veins  415.1 Pulmonary embolism and infarction  415.11 Iatrogenic pulmonary embolism  415.19 Other pulmonary embolism | 415.12 Septic pulmonary embolism  634.6x Spontaneous abortion complicated by embolism  635.6x Legally induced abortion complicated by embolism  636.6x Illegally induced abortion complicated by embolism  637.6x Unspecified abortion complicated by embolism  638.6 Failed attempted abortion complicated by embolism  639.6 Embolism not otherwise specified following abortion  673.2 Obstetrical blood-clot embolism  673.20 Unspecified as to episode of care or not applicable  673.21 Delivered, with or without mention of antepartum condition  673.22 Delivered, with mention of postpartum complication  673.23 Antepartum condition or complication  673.24 Postpartum condition or complication  673.3 Obstetrical pyemic and septic embolism  673.30 Unspecified as to episode of care or not applicable  673.31 Delivered, with or without mention of antepartum condition  673.32 Delivered, with mention of postpartum complication  673.33 Antepartum condition or complication  673.34 Postpartum condition or complication  671 Venous complications in pregnancy and the puerperium  671.3x Deep vein thrombophlebitis, antepartum  671.4x Deep vein thrombophlebitis, postpartum  671.9x Phlebitis, thrombosis not otherwise specified |

**^1^**Source: Moore, P.S., et al., *Trends in vena caval interruption.* J Vasc Surg, 2010. **52**(1): p. 118-125 e3; discussion 125-6.

**Appendix II-B. ICD Codes for Prior/Concurrent Comorbidities of Interest**

Individuals were identified as having one of the following conditions if they had at least one medical claim at any time during the 6-month pre-index period with the corresponding ICD-9-CM diagnosis code.

| **Condition** | **Code** |
| --- | --- |
| **Acute MI** | 410.xx |
| **Bleeding/Hemorrhage** | See Appendix C for diagnosis codes |
| **Cardiomyopathy** | 425.xx |
| **Cancer** | See Appendix D for diagnosis codes |
| **Coronary artery disease** | 411.xx, 412.xx, 413.xx, 414.xx, 429.2 |
| **Fracture of lower extremity** | 808.x, 821.xx, 823.xx |
| **Ischemic stroke** | 433.x1, 434.x1, 436.x |
| **Head Injury** | 800.xx, 801.xx, 803.xx, 804.xx |
| **TIA** | 435.x |
| **Heart failure** | 402.x1, 404.x1, 404.x3, 428.xx |
| **Hypertension** | 401.x, 402.x0, 403.xx, 404.x0, 404.x2, 405.xx |
| **Peripheral artery disease** | 440.xx, 443.xx |

Appendix II-C. ICD-9 Codes for History of Stroke and Bleeding

| **Medical Conditions** | **ICD-9 Code** | **Description** |
| --- | --- | --- |
| **Major Bleeding** | | Inclusive of hemorrhagic stroke, major intracranial bleeding and major extracranial bleeding below |
| **Hemorrhagic Stroke (Primary Only)** | 430 | Subarachnoid hemorrhage (SAH) |
|  | 431 | Intracerebral hemorrhage (ICH) |
|  | Exclusion | EXCLUDE above codes if “traumatic brain injury” ICD-9-CM code (800 to  804, 850 to 854) or “rehabilitation care” as the primary ICD-9-CM  code (V57) is present. |
| **Major Intracranial Bleeding**  **(Primary or Secondary Only)** | 430 | Subarachnoid hemorrhage |
|  | 431 | Intracerebral hemorrhage |
|  | 432.x | Other and unspecified intracranial hemorrhage |
|  | 852.0x | Subarachnoid hemorrhage following injury without mention of open intracranial wound |
|  | 852.2x | Subdural hemorrhage following injury without mention of open intracranial wound |
|  | 852.4x | Extradural hemorrhage following injury without mention of open intracranial wound |
|  | 853.0 | Other and unspecified intracranial hemorrhage following injury without mention of open intracranial wound |
|  | Exclusion | EXCLUDE – above codes if concomitant discharge diagnosis of major trauma was present (ICD-9 codes 852.1, 852.3, 852.5, and 853.1) |
| **Major upper GI bleed** | 531.0x | acute gastric ulcer with hemorrhage with/without obstruction |
|  | 531.2x | with hemorrhage and perforation with/without obstruction |
|  | 531.4x | (chronic or unspecified gastric ulcer with hemorrhage with/without obstruction) |
|  | 531.6x | (with hemorrhage and perforation with/without obstruction) |
|  | 532.0x | (acute duodenal ulcer with hemorrhage with/without obstruction) |
|  | 532.2x | (with hemorrhage and perforation with/without obstruction) |
|  | 532.4x | (chronic or unspecified duodenal ulcer with hemorrhage with/without obstruction) |
|  | 532.6x | (with hemorrhage and perforation with/without obstruction) |
|  | 533.0x | (acute peptic ulcer of unspecified site with hemorrhage with/without obstruction) |
|  | 533.2x | (with hemorrhage and perforation with/without obstruction) |
|  | 533.4x | (chronic or unspecified peptic ulcer of unspecified site with hemorrhage with/without obstruction) |
|  | 533.6x | (with hemorrhage and perforation with/without obstruction), |
|  | 534.0x | (acute gastrojejunal ulcer with hemorrhage with/without obstruction) |
|  | 534.2x | (with hemorrhage and perforation with/without obstruction) |
|  | 534.4x | (chronic or unspecified gastrojejunal ulcer with hemorrhage with/without obstruction) |
|  | 534.6x | (with hemorrhage and perforation with/without obstruction) |
|  | 578.0 | (hematemesis) |
|  | ICD-9 Procedure Code 44.43 | (endoscopic control of gastric or duodenal bleeding) |
|  | CPT code 43255 | (upper gastrointestinal endoscopy including esophagus, stomach, and either the duodenum and/or jejunum as appropriate with control of bleeding, any method) |
| **Major lower GI bleeding** | 562.02 | Diverticulosis of small intestine with hemorrhage |
|  | 562.03 | Diverticulitis of small intestine with hemorrhage |
|  | 562.12 | Diverticulosis of colon with hemorrhage |
|  | 562.13 | Diverticulitis of colon with hemorrhage |
|  | 569.3x | Hemorrhage of rectum and anus |
|  | 569.85 | Angiodysplasia of intestine with hemorrhage |
|  | 578.1x | Blood in stool |
|  | 578.9 | Hemorrhage of GI tract, unspecified |
| **Major urogenital bleed** | 599.7 | Hematuria |
|  | 626.2x and (280.0, 285.1 or 285.9) | Excessive/frequent menstruation and secondary diagnosis indicating acute bleeding (anemia) |
| **Other major bleeds** | 719.1x | Hemathrosis |
|  | 423.0x | Hemopericardium |
|  | 786.3x | Hemoptysis |
|  | 784.7x | Epistaxis |
|  | 459.0x | Hemorrhage not specified |
|  | 285.1x | Acute posthemorrhagic anemia |

*For algorithms requiring a primary discharge diagnosis from an inpatient setting, all primary diagnoses during an inpatient hospitalization were utilized to account for the lack of a well-defined discharge diagnosis in the Humana data.

Appendix II-D. ICD-9 Codes for History of Cancer

| **Major Group** | **Code** | **Malignant neoplasm of:** | **Major Group** | **Code** | **Malignant neoplasm of:** |
| --- | --- | --- | --- | --- | --- |
| **Head and Neck** | 140.xx | lip | **Gastrointestinal** | 150.xx | esophagus |
|  | 141.xx | tongue |  | 151.xx | stomach |
|  | 142.xx | salivary glands |  | 152.xx | small intestine |
|  | 143.xx | gum |  | 153.xx | colon |
|  | 144.xx | mouth |  | 154.xx | rectum |
|  | 145.xx | other parts of mouth |  | 155.xx | liver |
|  | 146.xx | ororpharynx |  | 156.xx | gall bladder |
|  | 147.xx | nasopharynx |  | 157.xx | pancreas |
|  | 148.xx | hypopharynx |  | 158.xx | retroperitoneum and peritoneum |
|  | 149.xx | ill defined sites of lip, oral cavity and pharynx |  | 159.xx | ill defined sites of digestive organs |
| **Respiratory Airway** | 160.xx | nasal cavities | **Bone, skin and breast** | 170.xx | bone and articular cartilage |
|  | 161.xx | larynx |  | 171.xx | connective and soft tissue |
|  | 162.xx | trachea, bronchus and lung |  | 172.xx | melanoma of skin |
|  | 163.xx | pleura |  | 173.xx | other malignancy of skin |
|  | 164.xx | thymus, heart and mediastinum |  | 174.xx | female breast |
|  | 165.xx | ill defined sites within respiratory system |  | 175.xx | male breast |
|  |  |  |  | 176.xx | Kaposi's sarcoma |
| **Genitourinary** | 179.xx | uterus, unspecified | **Central nervous system** | 190.xx | eye |
|  | 180.xx | cervix uteri |  | 191.xx | brain |
|  | 181.xx | placenta |  | 192.xx | other nervous system |
|  | 182.xx | body of uterus | **Endocrine** | 193.xx | thyroid |
|  | 183.xx | ovary |  | 194.xx | other endocrine |
|  | 184.xx | other female genital organs |  | 195.xx | other ill defined sites |
|  | 185.xx | prostate | **Recurrent or unknown** | 196.xx | secondary unspecified lymph nodes |
|  | 186.xx | testes |  | 197.xx | secondary of respiratory and digestive systems |
|  | 187.xx | penis and other male genital organs |  | 198.xx | secondary of unspecified sites |
|  | 188.xx | bladder |  | 199.xx | malignancy without specification of site |
|  | 189.xx | kidney and other urinary organs |  |  |  |
| **Hematology** | 200.xx | lymphoma | **Carcinoids** | 209.xx | neuroendocrine tumors and carcinoids |
|  | 201.xx | hodgkins lymphoma |  |  |  |
|  | 202.xx | other malignancy of lymphoid tissue | **Indeterminate** | 235.xx to 238.xx | neoplasms of uncertain behavior (not yet known if malignant or benign) |
|  | 203.xx | multiple myeloma |  |  |  |
|  | 204.xx | lymphoid leukemia |  |  |  |
|  | 205.xx | myeloid leukemia |  |  |  |
|  | 206.xx | monocytic leukemia |  |  |  |
|  | 207.xx | other leukemia |  |  |  |
|  | 208.xx | unspecified leukemia type |  |  |  |

Appendix II-E. HAS-BLED Bleed Risk Score

|  | **HAS-BLED Criteria** | **ICD-9-CM** | **HAS-BLED Score** |
| --- | --- | --- | --- |
| **H** | Hypertension | 401.x-405.x  Drugs used to treat hypertension (GPI codes 3300, 3400, 3610, 3615, 3620, 3625, 3630, 3640, 3660, 3720, 3760) | 1 |
| **A** | Abnormal renal and liver function (1 point for each) | Renal: 580.xx-586.xx, V56.0, V56.8; ICD-9 procedure codes 39.95, 54.98; CPT codes 90935-90993, 99512, 99559  Liver: 070.x, 155.0x, 155.1x, 155.2x, 571.x, 572.x, 573.x, 576.8, 456.0x, 456.1x, 456.2x; ICD-9 procedure codes 39.1x, 42.91 | 1 (renal) or 2 (liver) |
| **S** | Stroke | 433.xx-437.xx | 1 |
| **B** | Bleeding (includes anemia and other prior hemorrhage) | 285.0x, 285.1x, 285.9x, 423.0x, 430.x-432.x, 455.2x, 455.5x, 455.8x, 459.0x, 531.0x, 531.2x, 531.4x, 531.6x, 532.0x, 532.2x, 532.4x, 532.6x, 533.0x, 533.2x, 533.4x, 533.6x, 534.0x, 534.2x, 534.4x, 534.6x, 562.02, 562.03, 562.12, 562.13, 568.81, 569.3, 569.83, 569.85, 569.86, 578.0, 578.1, 578.9, 599.7x, 719.1x, 784.7x, 786.3x; ICD-9 procedure code 44.43; CPT code 43255 | 1 |
| **L** | Labile INRs^1^ |  | 1 |
| **E** | Elderly (age >65 years) | - | 1 |
| **D** | Drug or alcohol abuse (1 point for each) | Drug: NSAIDs^2^ (GPI^3^ codes 6410, 6610), Anti-platelets (8515), Other aspirin-containing products (3940990215, 4399100222, 4399100223, 4399100232, 4399100405, 4399400321, 4399400456, 4399590415, 4399590419, 6030990225, 6499000220, 6499000221, 6499000225, 6499000226, 6499000232, 6499000320, 6499000321, 6499000335, 6499000340, 6499000450, 6499000460, 6499100220, 6499100222, 6499100228, 6499100330, 6499100335, 6599000222, 6599100210, 6599100220, 6599100325, 6599100430, 6599100510, 6599170220, 6599400220, 7599000210, 7599000240, 7599000310, 7599000320, 8515990220, 9990000000)  Alcohol: 291.xx, 303.xx, 305.0x, 357.5x, 425.5x, 571.1x, 571.2x, 571.3x; ICD-9 procedure codes 94.61-94.63, 94.67-94.69 | 1 (drug), 1(alcohol) |
| **TOTAL SCORE** | | | 0-9^4^ |

^1^INR, International Normalized Ratio

^2^NSAIDs, nonsteroidal anti-inflammatory drugs

^3^GPI, Generic Product Identifier

^4^The maximum possible score was 8 since data were not available for labile INRs. The following risk classification categories were observed: low, 0-1; intermediate, 2; high, ≥ 3 (Wood EA, Malgor RD, Gasparis AP et al. Reporting the impact of inferior vena cava perforation by filters. *Phlebology*, 2014. 29(7): p. 471-5.)

**Appendix II-F. Geographic Regions**

| **Region** | **States** |
| --- | --- |
| Northeast | Connecticut, Massachusetts, Pennsylvania, New Hampshire, Rhode Island, New Jersey, Vermont, Maine, New York. |
| Midwest | Wisconsin, Michigan, Illinois, Indiana, Ohio, North Dakota, South Dakota, Nebraska, Kansas, Minnesota, Iowa, Missouri. |
| South | Alabama, Arkansas, District of Columbia, Florida, Georgia, Kentucky, Louisiana, Mississippi, North Carolina, South Carolina, Tennessee, West Virginia, Virginia, Oklahoma, Texas, Delaware, Maryland |
| West | Alaska, California, Colorado, Hawaii, Idaho, Montana, Nevada, Oregon, Utah, Washington, Wyoming, Arizona, New Mexico |

**Source:** http://www.census.gov/geo/www/geo_defn.html#GeographicCode

Appendix II-G. ICD-9 Codes for Anticoagulant Use

| **Class** | **Description** | **Generic Product Identifier**  **Code** | **HCPCS Code^1^** |
| --- | --- | --- | --- |
| Direct thrombin inhibitors | Dabigatran | 833370302001 |  |
|  | Argatroban | 83337015 | C9121 |
| Coumadin | Warfarin | 83200030 |  |
| Unfractionated heparin | Heparin | 83100020 | J1642, J1644 |
| Low-molecular-weight-heparin | Enoxaparin | 83101020 | J1650 |
|  | Tinzaparin | 83101080 | J1655 |
|  | Dalteparin | 83101010 | J1645 |
|  | Fondaparinux | 83103030 | J1652 |
| Direct Xa inhibitor | Rivaroxaban | 83370060 |  |
|  | Apixaban | 83370010 |  |

**^1^**HCPCS = Healthcare Common Procedure Coding System

**Appendix II-H. ICD-9 Codes for Device-Related Complications**

| 996.1 Mechanical complication of other vascular device, implant, and graft  996.62 Infection and inflammatory reaction due to other vascular device, implant, and graft  996.74 Other complications due to other vascular device, implant, and graft  999.2 Other vascular complications of medical care, not elsewhere classified  996.1 Mechanical complication of other vascular device, implant, and graft |
| --- |
